# Giant Direct Magnetoelectric Effect in Nanocomposite CoFe_2_O_4_:PVDF‐TrFE Thin Films

**DOI:** 10.1002/advs.77469

**Published:** 2026-09-03

**Authors:** Muireann de h‐Óra, Lynette Keeney, Rui Miguel Gonçalves Carvalho, Aliona Nicolenco, Tuhin Maity, Jordi Sort, Luís Amorim, Pedro Martins, Judith MacManus‐Driscoll

**Affiliations:** ^1^ Department of Materials Science and Metallurgy University of Cambridge Cambridge UK; ^2^ Tyndall National Institute, University College Cork Lee Maltings Complex, Dyke Parade Cork Ireland; ^3^ Physics Centre of Minho and Porto Universities (CF‐UM‐UP) and LaPMET – Laboratory of Physics for Materials and Emergent Technologies University of Minho Braga Portugal; ^4^ Departament de Física Universitat Autònoma de Barcelona Bellaterra Spain; ^5^ CIDETEC Basque Research and Technology Alliance (BRTA) Donostia‐San Sebastián Spain; ^6^ Indian Institute of Science Education and Research Thiruvananthapuram Thiruvananthapuram Kerala India; ^7^ Catalan Institute of Nanoscience and Nanotechnology (ICN2) CSIC and BIST Barcelona Spain; ^8^ Institució Catalana de Recerca i Estudis Avançats Barcelona Spain

**Keywords:** fabrication, ferroelectricity, magnetostriction, materials science, nanocomposite, nanomaterials, nanopillar, nanotechnology, piezoelectricity, thin film

## Abstract

Polymer‐based magnetoelectric (ME) materials are at the forefront of modern materials science. They have immense potential in a wide range of technological applications, including sensors, energy harvesters, and advanced memory and logic devices. Thus, the combination of magnetostrictive (MS) nanomaterials with lightweight flexible piezoelectric (PE) polymers enables the development of multifunctional systems suitable for versatile applications in the mechanical, electrical, and magnetic domains. Here, a novel three‐step fabrication process was employed to create a uniquely structured MS/PE nanocomposite. First, a vertically aligned nanocomposite (VAN) thin film of MS CoFe_2_O_4_ (CFO) nanopillars, self‐assembled in a benign MgO matrix phase, was grown. Then, wet chemical etching of the benign MgO phase was undertaken. Finally, the CFO nanopillars were coated with a ferroelectric (FE) polymer. The high interfacial area, highly crystalline nanocomposite system enabled efficient strain‐mediated coupling between the magnetic and electric order parameters. Thus, a significantly enhanced magnetoelectric coefficient ($\alpha_{33}^{DME}$) of 10 ± 1 V cm^−^^1^Oe^−^^1^ was achieved at room temperature, nearly two orders of magnitude higher than similar composite thin film systems previously reported. Our method offers a simple, versatile, and scalable approach to fabricating very high‐performance ME thin film devices.

## Graphical abstract

A novel three‐step grow‐etch‐fill fabrication approach creates structured cobalt ferrite/ferroelectric polymer nanocomposites with high interfacial areas. Driven by efficient strain‐mediated coupling, this crystalline system achieves a giant room‐temperature magnetoelectric coefficient nearly two orders of magnitude higher than other CFO/PVDF‐TrFE thin films. This scalable approach opens new pathways for high‐performance flexible electronics.

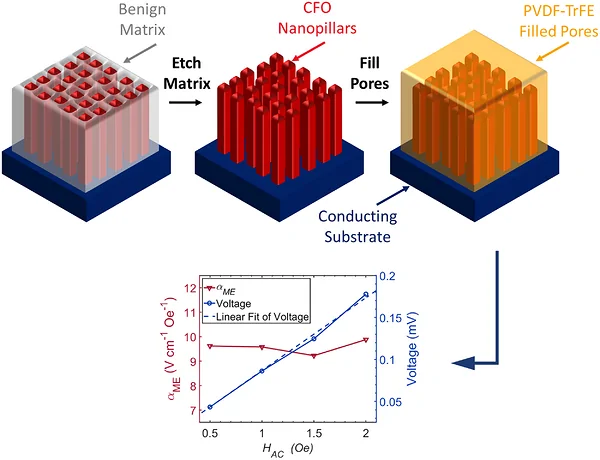

## 1 Introduction

Magnetoelectric (ME) materials possess either the ability to modulate electric polarization with magnetic fields, the direct ME effect (DME), or magnetization with electric fields [1, 2], the converse ME effect (CME). There has been extensive research on the materials for more than two decades owing to their large potential in a wide range of multifunctional devices [3, 4, 5, 6, 7]. Since the switching is not current‐driven, resistive losses are low, which means the energy efficiency is high [8, 9]. Applications of DME are very diverse [10, 11, 12, 13], but despite the strong demand for them, there are few demonstrations of high‐performing thin film devices.

Until now, most ME materials have been made from single‐crystal oxide thin film composite materials [14, 15, 16]. Single‐crystalline materials are often preferred for ME applications for several reasons: (i) enhanced ME coupling due to well‐defined crystal structures with long‐range order; (ii) reduced structural defects that can hinder the propagation of magnetostrictive (MS) or piezoelectric (PE) strains or can lead to electrical leakage; (iii) anisotropic properties allow for precise control and optimization of the ME response in specific crystallographic orientations, leading to improved performance; (iv) stability and reproducibility are achieved which ensures consistent performance, facilitating the integration of single crystalline ME materials into practical devices.

In all‐oxide composite thin films, electrical leakage is common, although some approaches have been successful in reducing this [12, 17]. Recent work on freestanding and heterogeneously integrated complex‐oxide systems has further demonstrated that releasing substrate clamping can significantly enhance strain transfer and magnetoelectric coupling, highlighting the importance of mechanically compliant architectures for high‐performance ME [18, 19, 20]. Polymer‐based composite materials, such as poly(vinylidene fluoride) (PVDF) co‐polymers, where magnetic materials are embedded in PE polymers, have been used to overcome leakage and to enable simple fabrication by spin‐coating [21]. Polymers are durable and flexible, allowing for high ME cyclability and coupling. They additionally open up possibilities for biomedical and flexible electronic devices [22]. The disadvantage of polymers is that the coupling coefficients (<1000 mV cm^−1^Oe^−1^) [23, 24, 25, 26] in polymer‐based devices are generally lower than single crystalline materials. Ongoing research, therefore, focuses on improving the ME performance of polymer composites through the incorporation of MS nanoparticles and by fabricating higher surface area interfacial structures. Despite the fact that multiferroic nanoparticle‐in‐film structures typically exhibit a smaller ME effect than planar structures, artificial multiferroic composites consisting of a polymer PE matrix and MS nanoparticles (3‐0 type) have achieved reasonably large ME effects [23].

Other forms of composite for achieving ME effects include planar (2‐2 type) layered thin film composites (most extensively studied), but they have the disadvantage that strain coupling is limited by substrate clamping [27, 28]. Vertically aligned nanocomposites (VANs, 3‐1 type) offer numerous advantages over conventional bulk and composite ME materials [29, 30, 31, 32]. VANs consist of a dense array of pillars within a matrix. Here, substrate clamping does not take place, and there is effective out‐of‐plane (OOP) strain transfer between the two phases in the composite over a large area [33]. Recent advances in CFO‐based nanostructures and vertically integrated oxide heterostructures have further demonstrated the importance of nanoscale strain engineering, interfacial coupling, and mechanically compliant architectures for enhancing magnetoelectric [20, 34]. There has been recent solid progress for the CME in all‐inorganic VAN films [17]. On the other hand, challenges of chemical, structural, and mechanical compatibility between phases in VANs (particularly inorganic‐organic ones) limit performance and applicability [35, 36].

Here we demonstrate the fabrication of novel 3‐D structured VAN films with MS CoFe_2_O_4_ (CFO) nanopillars coated with a polyvinylidene fluoride‐trifluoroethylene (PVDF‐TrFE) PE polymeric matrix, which has a giant DME. The strong performance is achieved by synergistic coupling between the two phases, where we exploit a large MS coefficient in CFO [37, 38, 39] through creating highly crystalline, vertically aligned nanopillars. These nanopillars are intimately strain‐coupled to the high PE coefficient, low elastic modulus PVDF‐TrFE matrix, with the large surface area of the 1–3 vertical architecture ensuring efficient vertical strain transfer between the MS and PE phases [40].

We use a novel 3‐step fabrication method to integrate the CFO with the polymer. Overall, we achieve films with DME coupling coefficient ($\alpha_{33}^{DME}$ = 10±1 V cm^−1^Oe^−1^) which is larger than any previously recorded CFO/PVDF‐TrFE based composite (α_DME_ = 0.15 V cm^−1^Oe^−1^) [24, 41] by nearly 2 orders of magnitude.

## 2 Method

**FIGURE 1:**
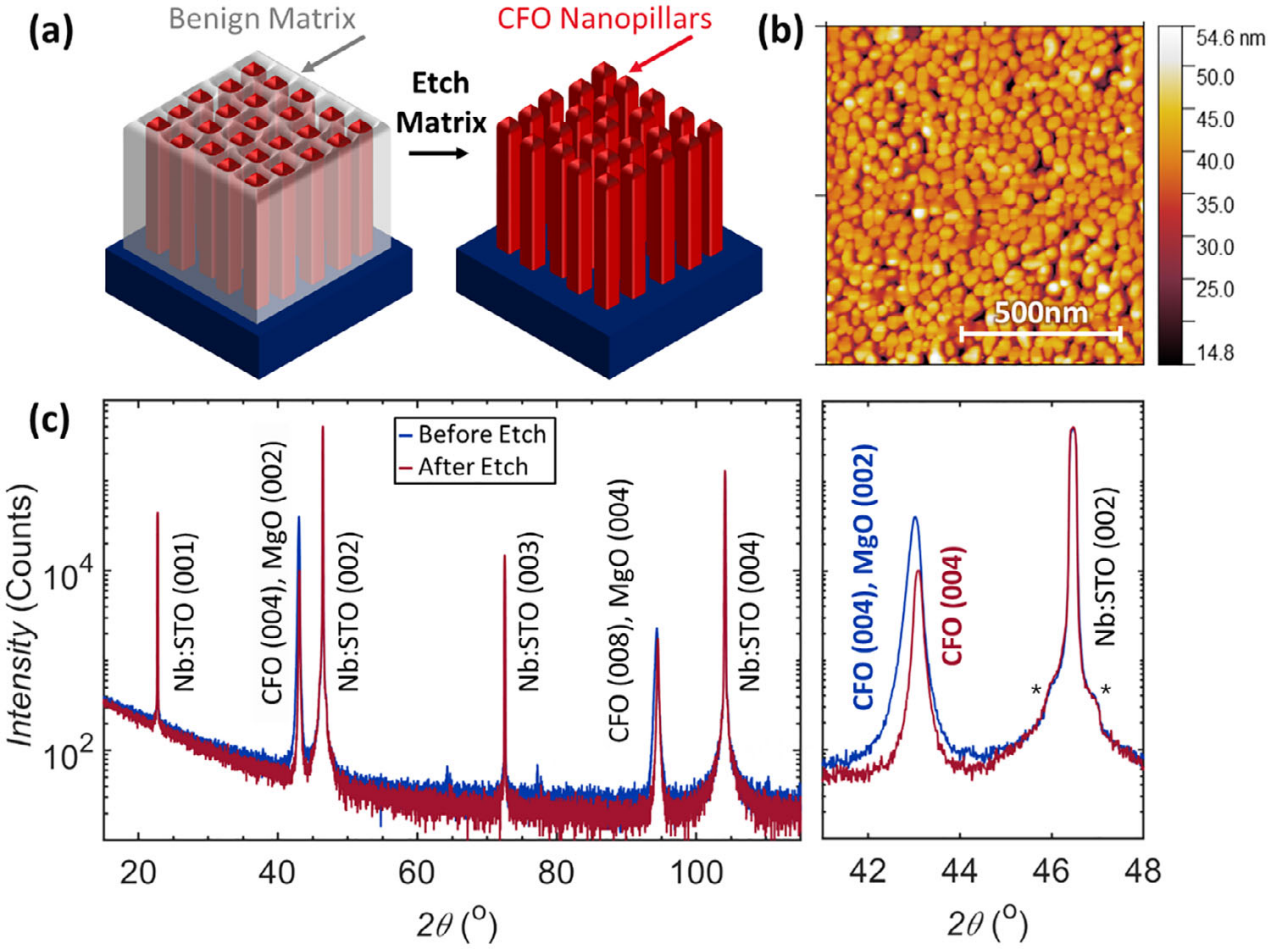
(a) Schematic of the growth of the VAN films of CFO nanopillars with a benign MgO matrix and the etching of the MgO. (b) AFM image of CFO nanopillars. (c) X‐Ray diffraction patterns for CFO:MgO composite before (blue) and after (red) the MgO was etched away. The CFO and MgO both grow (001) on the Nb:STO (001). The CFO retains its strain after the MgO is etched away, showing that it is not strained by the MgO.

In the first step (film growth), self‐assembled VAN films of MgO:CFO (CFO pillars in MgO matrix) are fabricated by PLD on 0.5 wt.% (weight percent) Nb‐doped SrTiO_3_ (Nb:STO) (001) substrates at 770°C in 0.1 Torr O_2_. A KrF laser (λ = 248 nm) at a fluence of 2 J cm^−2^ and a repetition rate of 5 Hz was used (Figure 1a‐left image). The PLD targets for the depositions were formed using solid‐state sintering from powders of Co_3_O_4_, Fe_3_O_4_, and MgO, followed by sintering at 1250°C for 6 h in air. The MgO to CFO ratio was 3:1 to ensure the correct MgO matrix and embedded CFO nanopillars.

In the second step, CFO free‐standing nanopillars (Figure 1b) were formed by wet‐chemically etching the MgO matrix from the VAN structure (Figure 1a ‐right image). This was done by submerging the sample in 10 wt.% (weight percentage) ammonium sulphate at 60°C for 2 h. The sample was continuously sonicated during the etching to ensure the complete removal of MgO from the VAN structure. The 30–60 nm diameter nanopillars form a dense vertical array.

**FIGURE 2:**
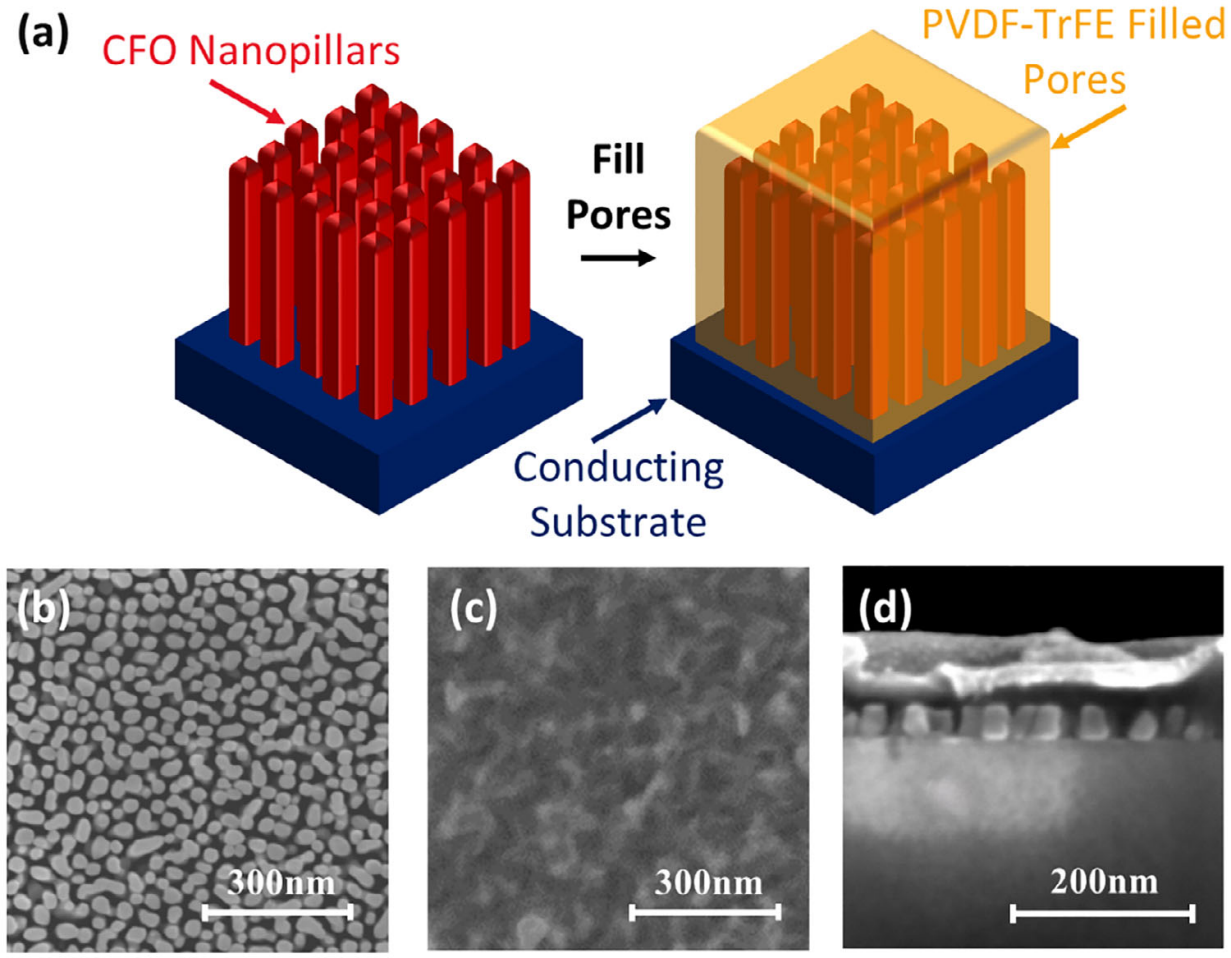
(a) Schematic of the etched VAN showing CFO nanopillars and the filling of the porous structure with PVDF‐TrFE. (b) SEM image of CFO nanopillars. The lighter regions of the SEM images represent the pillars, while the darker regions represent the etched matrix. (c) Top‐view SEM image of PVDF‐TrFE coating the nanopillar structure. (d) Cross‐sectional SEM image of CFO nanopillars hidden beneath PVDF‐TrFE, capped with a Pt electrode.

In the third step, the interpillar void regions were filled with PVDF‐TrFE (70‐30 ratio) by spin coating 40 µL of solution at 3700 rpm (Figure 2a). The dense CFO nanopillars guarantee that the PVDF‐TrFE fully penetrates the voids. The PVDF‐TrFE was then placed in a furnace at 125°C for 6 h to set. The PVDF‐TrFE copolymer was selected because its β‐phase has a polar crystal structure with permanent dipole moments arising from its chain conformation. These dipoles can be aligned by an electric field and remain oriented after its removal, a characteristic feature of FE behavior. The formation of the β‐phase is further accelerated by the nanoconfined environment of the 1–3 VAN structure [42]. The spatial constraints of the 20–40 nm pores force the polymer chains into the extended all‐trans conformation, effectively promoting the β‐phase during the filling process and optimizing the dipoles for subsequent alignment.

For structural characterization, X‐ray diffraction (XRD) patterns were recorded using a Panalytical Empyrean vertical diffractometer. Atomic force microscopy (AFM) was carried out using a Bruker Veeco Dimension Pro to examine the surface topography, while piezoresponse force microscopy (PFM) measurements were conducted using an Asylum Research (Oxford Instruments) MFP‐3D instrument to probe nanoscale electromechanical response. A FEI Nova FEG was used to conduct scanning electron microscopy (SEM). To achieve cross‐sectional images, samples were cleaved, mounted at an angle, and imaged using SEM. The film thickness was determined from the cross‐sectional SEM images using pixel‐based image analysis, where the PVDF‐TrFE and CFO regions were analysed to determine the average thickness and corresponding standard deviation across multiple points within the film (Figure S1).

ME measurements were conducted on the samples after sputter‐coating Au top electrodes of area 16 mm^2^ onto the devices. Prior to measuring DME effects, each sample was poled in a homemade corona chamber. The sample was placed approximately 2 cm from the corona grid and exposed to a 15 kV voltage at 110°C for 1 h. The samples were then cooled down to room temperature under the applied electric field.

Direct ME measurements were done using both a DC and AC magnetic field. A lock‐in amplifier was used to measure the change in voltage across the sample [43]. To determine the out‐of‐plane (OOP) ME coefficient ($\alpha_{33}^{DME}$), both DC (bias) and AC magnetic fields (*H*_DC_​ and *H*_AC_​) were applied simultaneously, aligned with the direction of the electric polarization in the PVDF‐TrFE composite film, which is oriented perpendicular to the film's surface. An AC magnetic field (maximum amplitude of 2 Oe, with frequencies varying from 1 Hz to 1 kHz) was generated using Helmholtz coils. A static bias magnetic field with a maximum strength of 1500 Oe was applied using an electromagnet. The lock‐in amplifier was referenced to the AC magnetic field frequency to ensure selective detection of the magnetoelectrically induced voltage and suppression of inductive and noise‐related artefacts. To verify that the measured response originated from the DME effect, the measurements were repeated using a depoled sample under identical experimental conditions.

The value of α_33_​ was then calculated using Equation (1):

(1) $$\alpha_{33}^{DME}=\frac{\Delta V_{3}}{d\Delta H_{3}}\;$$

where $\alpha_{33}^{DME}$ is the direct ME coefficient, *∆V_3_* is the out‐of‐plane measured voltage at the applied resonant frequency, *d* is the thickness of the composite system, and *∆H_3_* is the change in the applied AC magnetic field, H_AC_, out of plane.

## 3 Results and Discussion

### 3.1 Growth of CFO:PVDF‐TrFE Composites

As already noted, our unique FE polymer‐coated‐ MS nanopillar heterostructure was prepared using a three‐step grow‐etch‐fill process, where the MS material is first grown in a VAN structure alongside a benign material (Figures 1 and 2).

As shown in Figure 1c, the as‐grown VAN film is epitaxially aligned with (00l) peaks of CFO and MgO on the Nb‐STO (00l) substrate. After etching out the MgO, it is observed that the MgO (002) peak vanishes, indicating MgO removal. Furthermore, the CFO (004) peak remains unchanged in both intensity and peak position relative to the substrate, indicating no change in strain or microstructure in the CFO. An X‐ray reciprocal space map (Figure S2) supports this observation.

The microstructure of the PVDF‐TrFE‐coated CFO of Figure 2a (right image) was further examined. Figure 2b,c show SEM images of the free‐standing nanopillars and the continuous coating of the polymer deposited on the structure's surface, respectively. The spacing between the pillars is around 20–40 nm and is consistently reproduced across PLD growths (Figure S3). A cross‐sectional SEM of the coated nanopillars is shown in Figure 2d. The polymer (dark) penetrates the inter‐pillar spacing of the nanostructure (light), proving that the novel concept of the grow‐etch‐fill VAN structure could be fabricated. Insulating materials and polymers are difficult to image using SEM, but a clear contrast can be seen between the PVDF‐TrFE and the CFO. It can be seen in Figure 2c that the excess PVDF‐TrFE coats the top of the film's surface, to an overgrown thickness of ∼30 nm. This layer acts as an insulating barrier to lower electrical leakage throughout the system.

The fine lateral dimensions of the PVDF‐TrFE (embedded in the porous CFO structure) induce nanoconfinement of the polymer chains [42]. As already noted, this constraint forces a preferred orientation of the chains parallel to the vertical pillars, thereby optimizing the dipole alignment required for a high direct ME response. This alignment, combined with the polymer's low elastic modulus and the high crystallinity of the oriented CFO, ensures that a high degree of strain is efficiently imparted into the PVDF‐TrFE phase.

### 3.2 Ferroic Measurements

**FIGURE 3:**
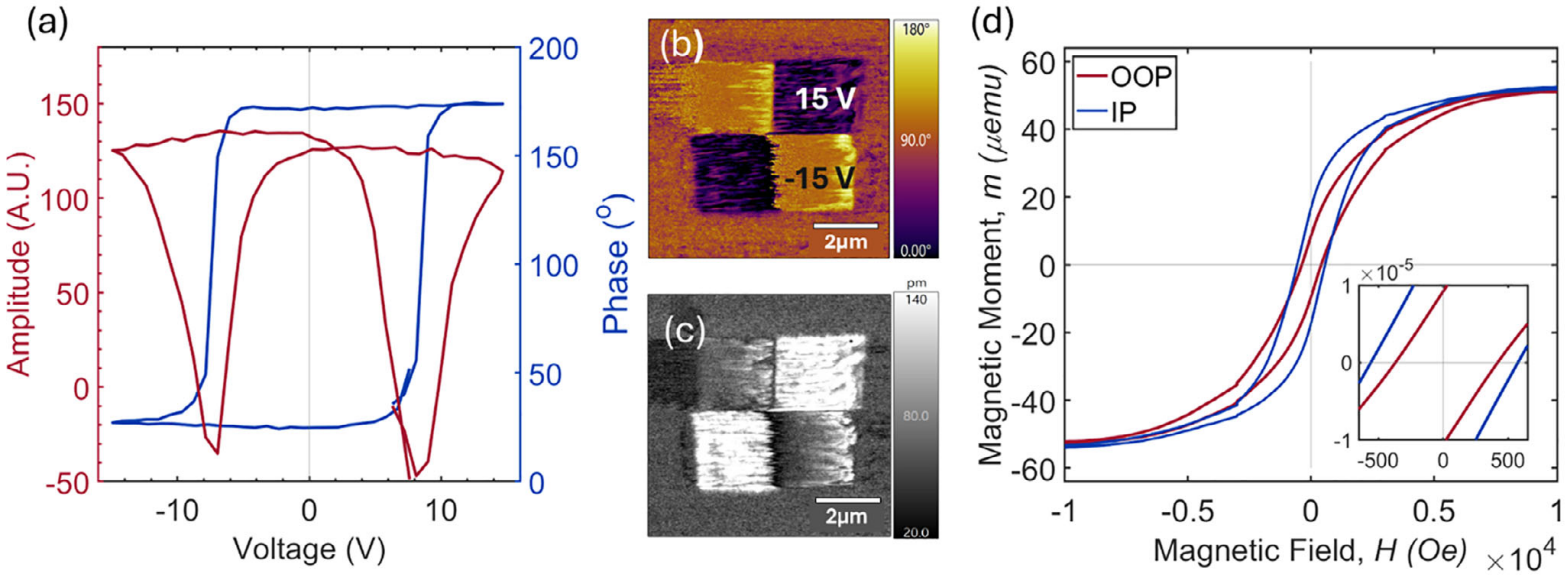
Ferroic characterization for PVDF‐TrFE deposited on CFO nanopillars. (a) PFM phase and amplitude loops showing the coercivity of the PVDF‐TrFE to be ∼8 V. (b) Phase and (c) amplitude checkerboard domain mapping of the PVDF‐TrFE surface after ferroelectric poling under applied DC biases of ±15 V, confirming the polymer's switchable ferroelectric behavior. (d) shows the magnetic hysteresis loops IP and OOP for the composite. The direct ME measurements were performed OOP, as the nanopillars are isotropic. Note that the artefacts in the loops at ±3 kOe were caused by the equipment.

The FE properties of the PVDF‐TrFE were examined as shown in Figure 3. Figure 3a displays PFM amplitude and phase hysteresis loops, confirming 180 ° switchable polarization with a coercive field of approximately −8 V.

Figure 3b,c show PFM phase and amplitude maps, respectively, after checkerboard poling with an applied DC bias of ±15 V and measured with a readout AC voltage of 3 V. This relatively low DC bias is sufficient to induce a full 180 ° phase shift in PVDF‐TrFE, as further evidenced by the representative phase and amplitude vs. voltage hysteresis loops in Figure 3a, a single example selected from measurements taken across a 32 × 32 point grid on the PVDF‐TrFE surface.

In the phase images (Figure 3b), the purple regions correspond to downward‐oriented ferroelectric domains written with a positive bias, while the yellow regions indicate upward‐oriented domains written with a negative bias. The same checkerboard pattern is evident in the corresponding amplitude map (Figure 3c). The checkerboard pattern appears slightly dragged along the scan direction, consistent with lateral deformation of the relatively soft polymer by the AFM tip in contact mode, although the underlying ferroelectric domain pattern remains intact.

Figure 3d displays the OOP and IP magnetic hysteresis loops. Both loops reveal a clear magnetic response of the CFO nanopillars, which is evident from the overall shape of the loops, with observable saturation magnetization typical of FM materials. Additionally, the loops exhibit a finite coercivity, H_C−IP_ = 565 Oe and H_C−OOP_ = 396 Oe, indicating that the pillars are not superparamagnetic and show semi‐hard FM behavior. The remanence‐to‐saturation magnetization ratio is larger for IP than OOP, suggesting a slight preferred orientation of the magnetization along the substrate plane (IP). The shape anisotropy is not more pronounced because of the relatively small aspect ratio of the pillars.

### 3.3 Magnetoelectric Measurements

**FIGURE 4:**
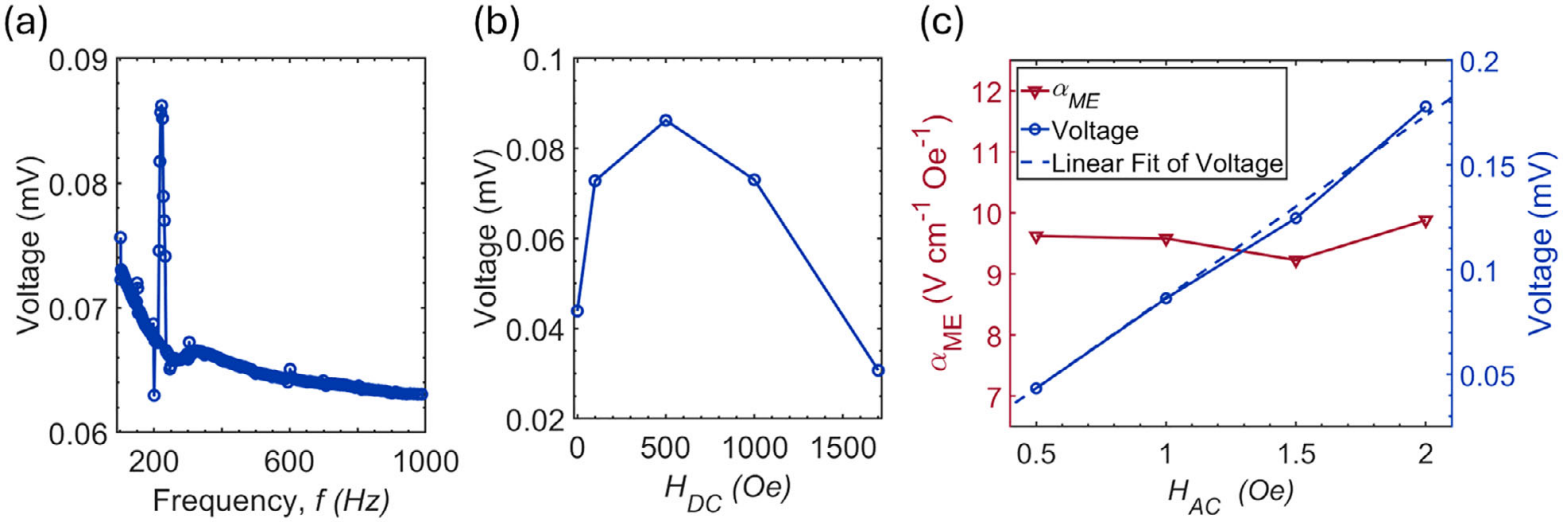
(a) The resonant frequency is 222 Hz. (b) Voltage output of the composite as a function of the applied DC magnetic field (H_DC_). H_DC_ = 550 Oe was used as a constant in the DME measurements as it yielded the largest voltage response. (c) Change in voltage of the composite relative to H_AC_ (right). The DME coefficient α_DM E_ was calculated from this data (left).

To measure the direct ME effect, both AC and DC magnetic fields were applied to the sample. An induced ME voltage was achieved upon field application. The calibration of the direct ME setup and the ME measurement of the CFO/PVDF‐TrFE composite are presented in Figure 4. Figure 4a shows a typical voltage–frequency response used to determine the resonant frequency of the CFO/PVDF‐TrFE composite, found to be 222 Hz. This resonance maximizes the ME output and reduces extraneous effects. It is set by the harmonic mode, composite thickness, in‐plane Young's modulus, volumetric mass density, and the mechanical boundary conditions imposed by the measurement configuration. This frequency was used for all subsequent measurements.

The optimal *H*_DC_ was determined from the voltage generated on the PVDF‐TrFE relative to the field, Figure 4b. The highest voltage was achieved at *H*_DC_ = 550 Oe (around the coercive field), at which the MS response of the material is maximized due to the increased strain induced at this magnetic field. The subsequent reduction in the ME voltage can be attributed to the saturation of the MS response in the CFO pillars. This calibration step is essential for obtaining the largest ME coupling in the films. The *H*_DC_ was held constant throughout the DME measurements.

Figure 4c presents the ME coupling within the CFO/PVDF‐TrFE VAN composite. The change in the induced voltage output of the PVDF‐TrFE relative to *H*_AC_ is shown in Figure 4c (right axis). A clear change in polarization is seen, even at low oscillating magnetic fields. The graph shows a linear effect, which is to be expected. As the applied magnetic field increases, the MS nanopillars elongate in the direction of the magnetic field, which strains the PE polymer and therefore increases its ferroelectric polarization.

Using Equation (1), the ME coefficient was calculated and is presented in Figure 4c (left axis). The largest $\alpha_{33}^{DME}$ = 10±1 V cm^−1^Oe^−1^ was found at *H*_AC_ = 2 Oe. This is the largest ME coefficient recorded for a CFO:PVDF‐TrFE composite system, and is nearly 2 orders of magnitude higher than previously reported CFO:PVDF‐TrFE nanoparticulate composites [$\alpha_{33}^{DME}$ = 150 mV cm^−1^Oe^−1^] [24, 41].

The large α_33_^DME^ arises from the highly crystalline, vertically aligned CFO nanopillars, which efficiently strain the FE PVDF‑TrFE across an extensive interfacial area. This high interface density is enabled by our novel fabrication method, which overcomes the usual limitations of polymer integration in VAN structures, specifically, the incompatibility of polymers with the high temperatures required for oxide growth. As a result, dense, fine PLD‑grown CFO nanopillars can be uniformly coated with PVDF‑TrFE, yielding the exceptionally large $\alpha_{33}^{DME}$ and demonstrating the effectiveness of our approach. Thus, the template, etched VAN film architecture not only enhances strain‐mediated coupling between the magnetic and electric phases but also offers more structural control compared to conventional film architectures, paving the way for even further enhancements of ME performance with nanostructure size tuning.

While a larger coupling coefficient ($\alpha_{33}^{DME}$ = ∼ 950 V cm^−1^Oe^−1^) has been reported in PVDF‐TrFE/Metglas foil in micron‐thick film composites [24, 25], due to massive bulk magnetostriction of Metglas (λ_s_  ≈  27 ppm [44]), compared to the value achieved here (PVDF‐TrFE:CFO nanopillars), our result obtained in a *thin‐film* structure provides critical advantages in structural scaling, biocompatibility, and potential for mechanical flexibility after substrate removal, features that are unattainable with brittle Metglas foils. Thus, our CFO:PVDF‐TrFE composite films are applicable for wearables and biomedical devices [45, 46]. Our system is also relevant to high‐density (CFO pillars have Tb/inch^2^ density) memory applications [47].

When compared to conventional 0–3 nanoparticulate CFO/PVDF‐TrFE systems [24, 41], our 1–3 architecture capitalizes on the exceptionally high vertical interfacial surface area inherent to the VAN design. In 0–3 particulate layouts, strain transfer is isotropic, severely limited by nanoparticle agglomeration, and dampened by the surrounding polymer matrix. By contrast, the continuous, anisotropic vertical interfaces of our vertically aligned CFO nanopillars establish direct 1–3 connectivity. This minimizes the shear‐lag effect, allowing the compressive/tensile strain of the pillars (λ_s_ ≈ −270 ppm intrinsically) [48] to couple directly to the active matrix along the film normal without the loss pathways typical of random particulate dispersions.

DME measurements in all‐oxide VAN systems are relatively scarce in the literature, largely reflecting the experimental challenges associated with reliably isolating intrinsic voltage signals and quantifying strain‐mediated coupling in these architectures. As a result, direct quantitative comparisons remain limited. Nevertheless, the available reports indicate DME responses of a similar order of magnitude to those observed in the present work [49, 50]. While traditional all‐oxide VANs [29, 30, 31, 32] inherently mitigate the severe substrate clamping that plagues conventional 2‐2 planar [27, 28, 33], there are constraints of the rigid oxide matrices that require high‐temperature processing and are prone to high leakage currents.

By transitioning to a hybrid oxide‐polymer VAN, using our grow‐etch‐fill method, we leverage the high mechanical compliance and elasticity of the PVDF‐TrFE matrix. This structural flexibility allows the high‐surface‐area CFO pillars to deform and transfer strain with significantly less mechanical resistance than an all‐oxide matrix would impose. The result is a unique VAN system that maintains the observed enhanced DME coefficient, while enabling low‐temperature fabrication and seamless integration potential with silicon electronics using epitaxial Nb:STO buffered Si [41].

To further assess the possible contribution of electrical leakage and electromagnetic artefacts to the measured magnetoelectric (ME) response, additional control experiments were performed and are provided in Figure S4. In particular, measurements were carried out on a depoled CFO/PVDF‐TrFE sample under identical experimental conditions, including variations in AC magnetic field frequency (1 Hz to 1 kHz) and DC magnetic bias (H_DC_). As shown in Figure S4a, the measured voltage exhibits a weak, nearly linear dependence on frequency, without any resonance features, and remains at low amplitude across the entire frequency range. Similarly, the voltage response as a function of H_DC_ (Figure S4b) shows no systematic dependence on the magnetic field.

These observations are inconsistent with a strain‐mediated magnetoelectric response and instead indicate a background signal unrelated to magnetoelectric coupling. Importantly, no enhancement at the resonance frequency (∼222 Hz) is observed in the depoled sample, confirming that the strong resonance behavior measured in poled samples is intrinsic to the magnetoelectric effect. Furthermore, the absence of a field‐dependent response in the depoled state rules out magnetic‐field‐induced leakage or inductive artefacts as dominant contributions to the measured signal. If such mechanisms were significant, a clear dependence on magnetic field amplitude or frequency would be expected.

In addition, the use of lock‐in detection referenced to the AC magnetic field frequency ensures that only voltage components strictly synchronous with the excitation are detected, effectively suppressing DC leakage currents and non‐correlated electrical artefacts. Taken together, these results confirm that the measured voltage response in poled samples originates from genuine strain‐mediated magnetoelectric coupling, and that the contribution of electrical leakage or inductive pickup to the extracted α_33_ coefficient is negligible.

## 4 Conclusions

A nanoengineered hybrid thin film structure was designed and fabricated to create a record, direct magnetoelectric coefficient, $\alpha_{33}^{DME}$ of 10±1 V cm^−1^Oe^−1^, 2 orders of magnitude higher than in conventional composites. High‐quality materials with superior properties *and* high interfacial area coupling between them enable the very high DME value. The coupled materials are comprised of highly aligned vertical CFO nanopillars (high anisotropy, high magnetostrictive coefficient, and high crystallinity) and PVDF‐TrFE (large piezoelectric coefficient, d_33_) coated around the CFO. Furthermore, the fine lateral spacing between the pillars induces nanoconfinement of the PVDF‐TrFE, which promotes the formation of the FE β‐phase and forces a preferred vertical orientation of the polymer chains. This alignment, combined with the high surface interface, allows the stiff CFO to efficiently strain the mechanically soft, flexible PVDF‐TrFE through the entire film thickness.

A 3‐step process was developed to enable the two materials to be combined in an optimal way: growth of a CFO:MgO VAN thin film structure, etching away of the sacrificial MgO matrix, and filling of the voids between the remnant CFO nanopillars with PVDF‐TrFE. Our novel process approach offers a versatile and scalable method for fabricating ME miniaturised devices, and it opens avenues for exploring a wider range of material combinations and devices with even higher DME values. Looking to the future, well‐established translatable processes for large‐scale manufacturing could be used to create the structures of this work. Hence, the nanopillars could be grown through techniques like sputtering or by chemical methods [13, 51, 52, 53], and an industry‐standard spin coating technique could be adopted for a PVDF‐TrFE coating step.

## Author Contributions

**Muireann de H‐óra**: conceptualization, investigation, writing – original draft, funding acquisition, methodology, validation, writing – review and editing, formal analysis, data curation, visualization, project administration. **Lynette Keeney**: investigation, methodology, Writing – review and editing, formal analysis, software. **Aliona Nicolenco**: investigation, methodology. **Jordi Sort**: writing – original draft, writing – review and editing, conceptualization, methodology, validation, supervision. **Luís Amorim**: supervision. **Rui Miguel Gonçalves Carvalho**: investigation, methodology. **Tuhin Maity**: writing – review and editing, writing – original draft. **Pedro Martins**: supervision, investigation, validation, methodology. **Judith Macmanus‐driscoll**: funding acquisition, conceptualization, methodology, validation, writing – review and editing, writing – original draft, supervision, resources, visualization.

## Conflicts of Interest

The authors declare no conflicts of interest.

## Supporting information




**Supporting File**: advs77469‐sup‐0001‐SuppMat.docx.

## Data Availability

The data that support the findings of this study are available from the corresponding author upon reasonable request.
