# Supplementary material for: Giant Direct Magnetoelectric Effect in Nanocomposite CoFe2O4:PVDF‐TrFE Thin Films
Source: Adv Sci (Weinh). 2026 Sep 3:e77469. Online ahead of print. doi: 10.1002/advs.77469 (PMC13542401; doi:10.1002/advs.77469)
Supplement: Supplementary file 1 [file ADVS-9999-e77469-s001.docx]

*Figure S1. (a) Cross-sectional SEM image of CFO nanopillars embedded beneath the PVDF-TrFE layer and capped with a Pt electrode. An overlay of the image processing used to isolate the PVDF-TrFE region is shown in black and is further presented in (b). (b) Processed SEM image used for thickness and height analysis. (c) Height-filter analysis extracted from image (b) corresponding to the PVDF-TrFE thickness distribution. (d) Height analysis of the CFO nanopillars obtained from the processed SEM image.*

Image analysis of the CFO nanopillars was performed using a height range of 42–80 nm, yielding an average pillar height of 58.8 nm with a standard deviation of 6.1 nm across 1424 selected points. For the PVDF-TrFE layer, and thus total thickness of the film, a height range of 40–140 nm was used, resulting in an average thickness of 89.8 nm with a standard deviation of 10.7 nm across 2388 valid points.

*Figure S2 (a) Reciprocal space map around the CFO (226) peak before and (b) after the.*

*MgO is etched away. The MgO (113) and CFO (226) peaks overlap so when the MgO is*

*etched away, the peak intensity decreases, but the peak does not shift.*

A reciprocal space map (RSM) was done around the CFO (226) peak before and after etching

to see how the strain changes. The peak before etching (Figure S1 (a)) is strong as it overlaps with the MgO (113) peak. This peak decreases when the MgO is etched away, as the film has higher MgO content, but the position of the peak remains the same, indicating no relaxation in the CFO. The STO peak has also decreased in intensity owing to the fact that the smaller CFO (226) peak had to be more carefully aligned.

*Figure S3: SEM images from different batches of CFO nanopillars formed from etching VAN films showing the reproducible structures achieved from batch to batch.*

*Figure S4: (a) Measured voltage response of a depoled CFO/PVDF-TrFE sample as a function of AC magnetic field frequency (1 Hz–1 kHz) under identical experimental conditions. The voltage exhibits a weak, approximately linear dependence on frequency, with no observable resonance features and consistently low amplitude across the measured range. (b) Measured voltage response as a function of DC magnetic bias field, H_DC_, showing no systematic dependence on the applied magnetic field.*
